# Comprehensive Epstein-Barr Virus Transcriptome by RNA-Sequencing in Angioimmunoblastic T Cell Lymphoma (AITL) and Other Lymphomas

**DOI:** 10.3390/cancers13040610

**Published:** 2021-02-04

**Authors:** Nader Bayda, Valentin Tilloy, Alain Chaunavel, Racha Bahri, Mohamad Adnan Halabi, Jean Feuillard, Arnaud Jaccard, Sylvie Ranger-Rogez

**Affiliations:** 1 Microbiology Department, UMR CNRS 7276, INSERM U1262, Faculty of Pharmacy, 87025 Limoges, France; nader.bayda@unilim.fr (N.B.); racha_bahri91@outlook.com (R.B.); adnan.halaby@gmail.com (M.A.H.); 2 Department of Infectious Disease Control, Faculty of Public Health, Jinan University, Tripoli 1300, Lebanon; 3 National Reference Center for Herpesviruses, Bioinformatics, Centre de Biologie Recherche et Santé, 87000 Limoges, France; valentin.tilloy@unilim.fr; 4 Pathology Department, Centre de Biologie Recherche et Santé, 87000 Limoges, France; alain.chaunavel@chu-limoges.fr; 5 Biological Hematology Department, UMR CNRS 7276, INSERM U1262, Centre de Biologie Recherche et Santé, 87000 Limoges, France; jean.feuillard@unilim.fr; 6 Clinical Hematology Department, UMR CNRS 7276, INSERM U1262, University Hospital Dupuytren, 87042 Limoges, France; arnaud.jaccard@unilim.fr; 7 Virology Department, UMR CNRS 7276, INSERM U1262, Centre de Biologie Recherche et Santé, 87000 Limoges, France

**Keywords:** transcriptome, transcripts, Epstein-Barr virus, EBV, angioimmunoblastic T cell lymphoma, AITL, lymphoma, next-generation sequencing, NGS

## Abstract

**Simple Summary:**

Angioimmunoblastic T cell lymphoma (AITL) is probably the most common peripheral T-cell lymphoma. This pathology, although rare, is more common in Europe than in other regions. This lymphoma has a poor prognosis. AITL is very commonly associated with the Epstein-Barr virus (EBV) although the virus is not often found in neoplastic T cells but rather in adjacent B cells. Our objective was to study the transcriptome of EBV in AITLs comparatively to other EBV-associated lymphomas and to compare the results with those obtained for cell lines. We showed in AITLs a strong expression of Bam-HI A rightward transcripts (BARTs) more expressed than in the other lymphomas and especially than in cell lines. BARTs can participate in tumor development. We also showed a latency IIc in AITLs with the expression of *BNLF2a* and *BCRF1* genes which may participate in the survival of infected cells. These results support the involvement of EBV in AITLs.

**Abstract:**

The Epstein–Barr virus (EBV) is associated with angioimmunoblastic T cell lymphoma (AITL) in more than 80% of cases. Few studies have focused on this association and it is not clear now what role the virus plays in this pathology. We used next-generation sequencing (NGS) to study EBV transcriptome in 14 AITLs compared to 21 other lymphoma samples and 11 cell lines including 4 lymphoblastoid cell lines (LCLs). Viral transcripts were recovered using capture probes and sequencing was performed on Illumina. Bam-HI A rightward transcripts (BARTs) were the most latency transcripts expressed in AITLs, suggesting they may play a role in this pathology. Thus, BARTs, already described as highly expressed in carcinoma cells, are also very present in AITLs and other lymphomas. They were poorly expressed in cell lines other than LCLs. AITLs showed a latency IIc, with *BNLF2a* gene expression. For most AITLs, *BCRF1*, which encodes a homologous protein of human interleukin 10, vIL-10, was in addition expressed. This co-expression can contribute to immune escape and survival of infected cells. Considering these results, it can be assumed that EBV plays a pathogenic role in AITLs.

## 1. Introduction

The Epstein–Barr virus (EBV) is a widespread human gamma-herpesvirus of which two types can be distinguished, EBV type 1 (EBV-1) and EBV type 2 (EBV-2), according to geographic distribution, virulence, and differences in the latent genes (principally *Epstein*–*Barr nuclear antigen-2 (EBNA-2)*, *-3A*, and *-3C* genes) [1]. EBV prevalence is very high and almost all adults have been infected [2]. As with all herpesviruses, the primary infection is followed by a lifelong latency defined by the absence of production of new infectious virions. In the event of infected cell activation, episodes of viral reactivation may occur, corresponding to a resumption of the lytic cycle with the production of infectious virus [3].

First resting B-cell EBV infection leads to expression of the Epstein–Barr nuclear antigen-2 (EBNA-2) and EBNA-Leader Protein (EBNA-LP) proteins as well as latent BHRF1, a bcl2 homolog protein, driven by the activated viral promoter Wp [4]. The upstream viral promoter Cp is then activated by both expressed EBNAs and the cellular factor recombination signal binding protein for the immunoglobulin Kappa J region (RBP-Jκ), leading to the production of the six EBNA proteins, EBNA-1, EBNA-2, EBNA-3A, -3B, -3C, and EBNA-LP. The Wp promoter is gradually hypermethylated and transcription passes under Cp control [5]. At the same time, the latent membrane proteins LMP-1, LMP-2A, and LMP-2B are produced following the activation of their promoters. *Bam*-HI A rightward transcripts (BARTs) and non-coding RNAs, Epstein–Barr virus-encoded small RNAs (EBERs), are also transcribed, plus a set of miRNAs. This latency pattern, defined as latency III (Lat III), drives B cell growth transformation resulting in the establishment of permanent in vitro lymphoblastoid cell lines (LCLs). Lat III is found in immunocompromised lymphomas.

Other latency patterns have also been described in which the latency gene expression is more restricted [6,7,8]. Latency 0 (Lat 0), characterized by the presence of EBER, BART, miRNAs, and possibly LMP-2A transcripts, in the absence of any other EBV protein, is found in resting recirculating memory B cells of healthy subjects. Latency I (Lat I), identified in Burkitt’s lymphoma (BL) biopsies and in lines derived from BL, is characterized by the expression of a single viral protein, EBNA-1, and the production of non-coding RNAs. In this form of latency, the Wp and Cp promoters are inactive and the production of the EBNA-1 protein is solely dependent on the alternative viral promoter Qp. Lat I is also observed during cell division in memory B cells. In latency II (Lat II), activation of the Qp promoter also results in the production of EBNA-1, but at the same time, LMP-1, LMP-2A, and LMP-2B are also expressed as the EBERs, BARTs, and miRNAs. Lat II is described in NK/T lymphoma (NK/TL), Hodgkin’s lymphoma (HL), and nasopharyngeal carcinoma (NPC) tumors [9]. Only a small number of viral genes are therefore involved in any latency. These different forms of latency have thus been well characterized in B lymphocytes, but a continuum likely exists in vivo between these different models, and it was suggested that expression of latency proteins varies with cell differentiation [10]. Recent studies have reported a brief period after infection and before cell division during which there is a transient explosion of lytic gene expression, without viral replication, and concomitantly with the expression of the first latent genes from the Wp promoter [10,11,12,13]. This latency phase, characterized by the presence of EBNA-2 in the absence of LMP-1, was named the pre-latent phase or “latency IIb”. It differs from latency IIa which follows latency III and is defined by the presence of LMP-1 in the absence of EBNA-2. Furthermore, it is known that EBV can also establish a latent infection of epithelial cells or NK/T lymphocytes, but the conditions are less well known.

In contrast to the latent state, entering the lytic cycle corresponds to the activation of more than 80 genes [14]. Gene expression is coordinated over time and the activation of the very early transactivators BZLF1 and BRLF1 induces the expression of the immediate early (IE) genes, leading to the expression of early (E) genes, including those necessary for genome replication followed by late (L) genes which encoding structural proteins [15,16].

EBV is involved in various B cell malignancies, such as endemic BL where it is found in about 80% of cases [17], HL with 30% of cases associated with the virus, especially mixed cellularity classical HL (MC-cHL), and more rarely immunoblastic lymphomas occurring in immunocompromised patients. The oncogenic role of EBV, and in particular the role of the different latency proteins, is now well established [18]. EBV is also implicated in carcinomas such as NPCs where it is always present, or gastric carcinomas (GCs) where it is detected in approximately 10% of cases [18]. Peripheral T-cell lymphomas (PTCLs), which are uncommon pathologies since they represent 15% of non-Hodgkin’s lymphomas (NHLs), are occasionally associated with EBV. For example, in extranodal NK/T-cell lymphomas, the virus, detected in 71% of cases, infects tumor cells where it is constantly detected. Angioimmunoblastic T cell lymphoma (AITL), which is the focus of our work, is probably the most common form of PTCL [19] although less common in North America or Asia than in Europe, accounting respectively for 16–34.4%, 17.9–22.4%, and 28–34% of PTCLs [20,21]. AITL, which mostly affects elderly people [22], is clinically characterized by generalized lymphadenopathy often accompanied by hepatosplenomegaly, skin rash, and B-cell modifications associated with immunologic abnormalities. This is an aggressive lymphoma with a poor prognosis. Pathologic findings reveal abolished lymph node architecture with an extensive polymorphous inflammatory infiltrate including EBV-positive B-cells adjacent to neoplastic cells of T follicular helper origin (Tfh). Increased numbers of follicular dendritic cells are observed near abundant arborescent endothelial venules. EBV is detected in 85–95% of AITL, being most often present in large B blasts, but sometimes described in neoplastic T cells [23,24,25,26,27]. It has been proposed that AITL generates an immunodeficiency at the origin of EBV reactivation promoting the expansion of Tfh and B cells, thus playing a role in the development of the tumor microenvironment. Or EBV itself could induce AITL development by activating Tfh cells [28] unless the interaction between B lymphocytes and neoplastic cells provides support for tumor development [29]. Depending on the hypotheses considered, the mechanisms are different.

For these reasons, in this study, we examined the EBV transcriptome in 14 AITL biopsies compared to other EBV positive lymphomas and cell lines by RNA-seq, We demonstrated for AITLs a significant expression of BARTs superior to that observed for other lymphomas. In addition, we reported that AITLs exhibited latency IIc with a strong expression of *BNLF2a*. A group of six late genes was also expressed (*BCRF1*, *BSRF1*, *BVRF1*, *BNRF1*, *BFRF3*, and *BOLF*).

## 2. Results

### 2.1. Analysis of EBV Gene Expression by New Generation Sequencing (NGS)

We performed massive parallel sequencing by NGS after mRNA enrichment then EBV transcripts capture, to evaluate the EBV transcriptome in 11 EBV positive human lines, which included 4 LCLs, and 35 EBV positive lymphomas from patients, including 14 AITLs, selected based on EBER positivity by *in situ* hybridization (ISH). Unfortunately, we did not have enough material to study which cells carried the virus. NGS results are shown in Table 1; mean read number per sample was 2,477,173 (from 600,000 to 10,066,666) with a depth comprised between 42 and 2,010 (mean depth 378). Data were submitted to SRA and are available under BioProject ID PRJNA686869. As could be expected, all patient samples were EBV type 1. EBV type 1 is more prevalent and also more virulent than type 2 EBV, which is mainly confined to the African continent.

### 2.2. AITL Samples Are Not Homogeneous

**Figure 1:**
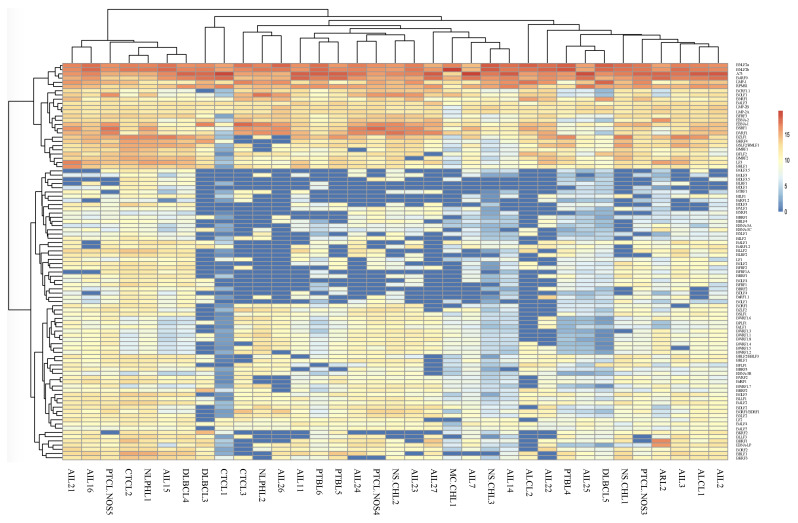
Heatmap of EBV gene expression for the 14 angioimmunoblastic T cell lymphoma (AITL) and 21 other EBV-associated lymphomas.

Analysis of results for all patients showed that samples from the 14 AITL patients did not form a cluster (Figure 1). The same was also found for the other pathologies, although the number was low in each case. It was notable that a group of patients, comprising various pathologies (3 AITL, 1 PTCL-NOS, 1 CTCL, 1 NLPHL, and 1 DLBCL), expressed a much larger variety of different viral genes than the others. It can also be noticed that a group of genes including latency genes (*EBNA-1*, *LMP*s, *EBNA-2*, and BARTs), and 17 other genes were expressed for the majority of patients while the other genes were only slightly or not expressed at all.

### 2.3. BARTs Are the Most Abundant Latency Transcripts in Lymphoma Samples

We analyzed the viral transcripts present in samples and, because we performed a poly(A)+ capture before sequencing, we did not detect any non-polyadenylated transcripts, for example, the EBERs.

**Figure 2:**
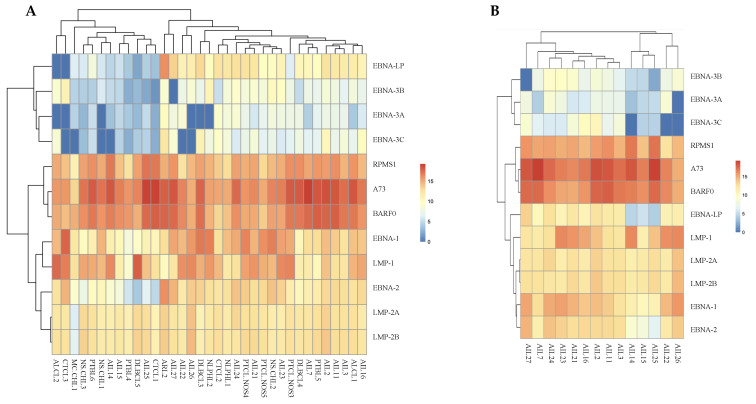
EBV latency gene expression analysis. (**A**) A heatmap shows results obtained for all patient samples tested. (**B**) Heatmap of latency transcripts for AITLs.

**Figure 3:**
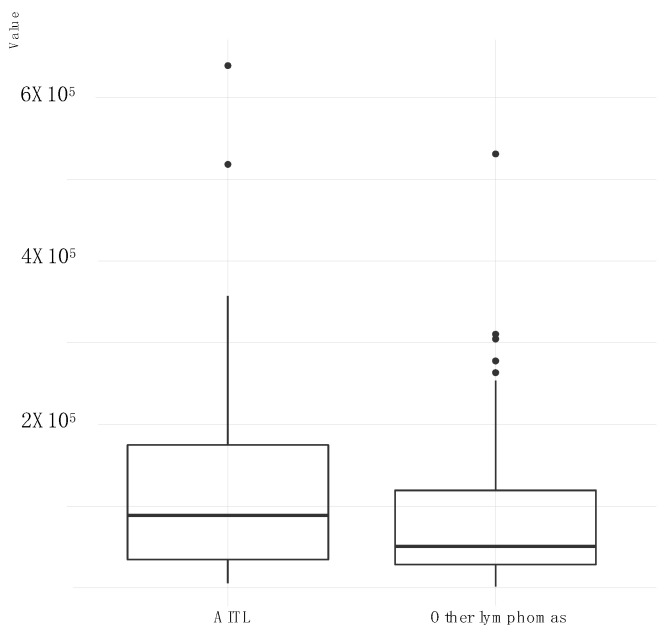
Distribution of BARTs (BARF0, A73, RPMS1) genes expression for AITL lymphomas comparatively to other lymphomas. For each group, a boxplot is represented showing the lower and upper quartile (the 25th and 75th percentiles, respectively). Line inside boxplot shows the median whereas vertical lines outside the box represent the minimum and maximum (0th and 100th percentile) excluding outliers.

For AITLs, the results obtained show that the most expressed latency transcripts are BARTs (Figure 2B), which was much less frequent for other patients (Figure 2A and Figure 3). The BARTs constitute a complex set of differentially spliced polyadenylated RNAs that share the same 3′ end and originate from several putative open reading frames, namely BARF0, RPMS1, and A73 [30]. Our results showed increased expression of A73 and BARF0 respective to RPMS1 in AITLs.

### 2.4. All AITL Samples Tested Are in Latency II but Show Strong Expression BNLF2a

**Figure 4:**
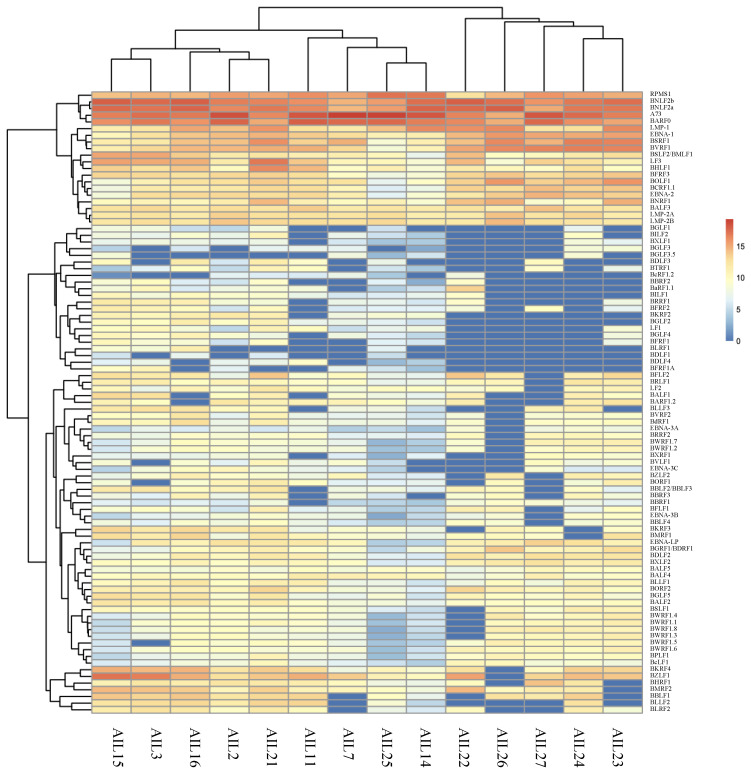
Heatmap of all EBV gene expression for AITLs.

AITL biopsies and more largely all patient samples tested carried latency II EBV as evidenced by the lack of EBNA-3 expression (Figure 2A). Nevertheless, all but 3 AITL contained EBNA-2 and EBNA-LP transcripts. Moreover, expression of the viral transcription factor BZLF1 was present with weak expression of BRLF1 and lack of expression of all proteins of the lytic cycle (Figure 4). However, it is very remarkable that some lytic genes were expressed especially the early gene *BNLF2a*. Among the lytic genes, it seems that six of them, the late genes *BCRF1*, *BSRF1*, *BVRF1*, *BNRF1*, *BFRF3*, and *BOLF1*, present a clustered expression in the majority of AITLs. Interestingly, BCRF1 encodes for a protein that shows great homology with human interleukin-10 (IL-10) and is referred to as v-IL-10.

### 2.5. Cell Line Sequencing

We studied the EBV transcriptome in B95-8 line, initially obtained from a primary infection, in four BL lines (Jijoye, Namalwa, P3HR1, and Raji), in two NK/TL lines (SNK6 and MEC04), characteristics of which are mentioned in Table 2 and in four LCL that we established (CoAN, DPL, KREB2, and MLEB2).

**Figure 5:**
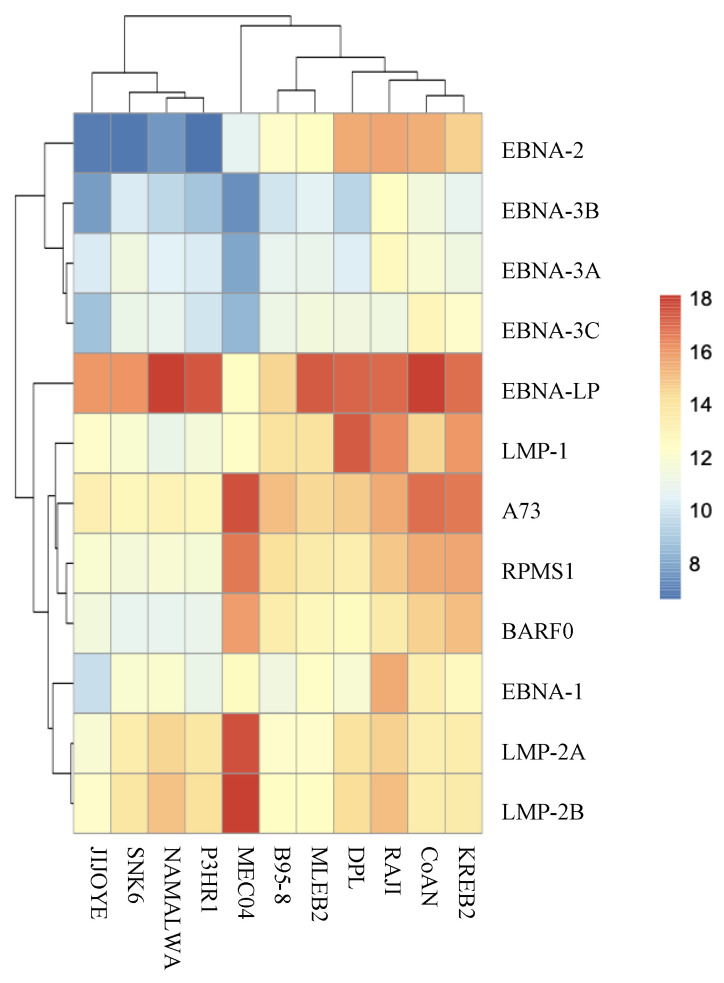
EBV gene expression analysis in the cell lines studied: heatmap of latency transcripts.

It may be noted that the cell lines expressed little or no EBNA-3 and that only LCL and Raji expressed EBNA-2 (Figure 5). BARTs were much more present in LCLs than in other cell lines, except MEC04. It is noteworthy that, apart from DPL, cell lines clustered and behaved homogeneously regardless of their origin. Among the genes most expressed in the lines is *BHRF1* which codes for a bcl2 homolog and miRNAs. EBNA-LP was also highly expressed in all cell lines except MEC04. It can be noticed that MEC04 has a very different profile from other lines.

**Figure 6:**
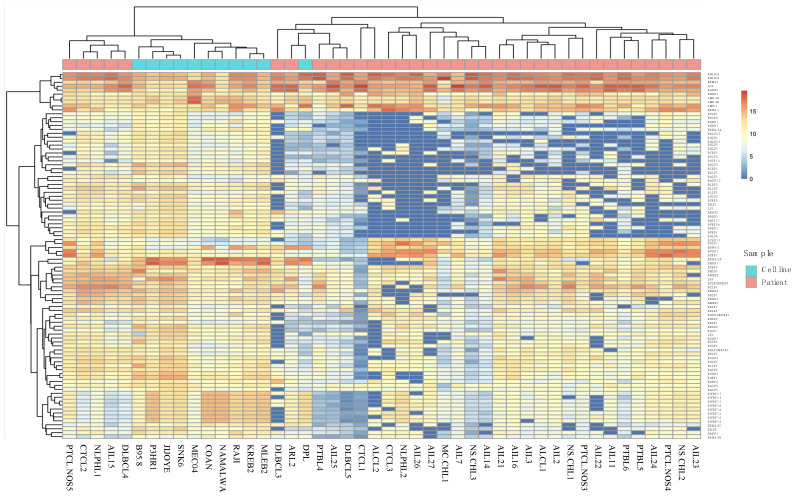
Heatmap of EBV gene expression for the patients and the cell lines.

The deletions described for some lines resulted, when they affect the entire gene, in a lack of expression particularly clearly visible in the case of *LF1*, *LF2*, and *LF3* for B95-8 or *EBNA-2* for P3HR1 (Figure 6).

## 3. Discussion

In this study, we wanted to assess the expression level of viral transcripts in AITL in comparison to some lymphadenopathies from other lymphomas. Cell lines were also used as a comparative element as the type of latency they carry has already been established.

Overall, there was no obvious difference between AITLs and other lymphomas and there was no clustering of AITLs. Gene expression varies according to lymphoma and even between tumors of the same pathology. Interestingly, there was no complete lytic cycle among AITLs, therefore no viral reactivation which makes it possible to produce new infectious particles.

RNAseq analysis assessed the respective quantity of transcripts and revealed that among the latency transcripts, the BARTs were very largely predominant. BARTs were expressed in very high amounts in all AITLs and more broadly they were also found in abundance in other patients. BARTs were initially demonstrated in NPC xenografts [51] as well as in NPC biopsies [52] and also in BL biopsies [53]. Verhoeven et al. [54] described high BART expression in NPC and discussed their role in this pathology. BARTs have also been highlighted in GCs [55] where their strong expression could contribute to growth regulation and would therefore constitute a mechanism of viral oncogenesis [56]. Thus, it has been gradually accepted that BARTs are very strongly expressed in infected NPC and GC epithelial cells, probably participating in these pathologies. Our findings show that BARTs were not only expressed in EBV epithelial malignancies but also at high levels in lymphomas and especially in AITLs. Most probably they are present in any form of cancer as suggested by Chakravorty et al. [17]. BARTs have been described as expressed in all latency phases, although predominantly during latencies I or II [57], and also during the lytic cycle. Although the putative proteins encoded by BARTs show potentially significant properties with regards to cell transformation [51], they never have been detected in vivo [30,58]. Since a nuclear localization of these persistent RNAs has been reported, it has been suggested that they function as long non-coding RNAs (lncRNAs) that selectively regulate viral and/or cellular gene expression [54]. BARTs also encode mature intronic micro RNAs (miRNAs) many of which are expressed at a high rate in the same tumors which carry BARTS. These miRNAs mainly contribute to tumor development or growth by ensuring the maintenance of latency by blocking lytic transcripts, and also by blocking the immune defenses [59,60]. The fact that BART transcripts were found in abundance in our AITL patients suggests that they play an important role in this pathology, whether they act in the form of lncRNAs and/or miRNAs.

In this study, it is remarkable that the *BNLF2a/BNLF2b* genes, described as early lytic genes, are strongly expressed in patient samples and especially for AITLs; they are even the most expressed genes (Figure 4). In the AITL biopsies we tested, the virus could be considered to be in latency II due to the absence of EBNA-3 expression. However, the expression of EBNA-2, and to a lesser extent EBNA-LP, in at least 11 out the 14 tissues tested, does not support a latency IIa state. This was also found in 57.1% of other patients. Moreover, the simultaneous LMP-1 expression excludes the hypothesis of latency IIb. We wondered if the virus was starting to reactivate. Resumption of a lytic cycle begins with the induction of viral transcription factors, particularly BZLF1 and BRLF1, and activation of viral promoters. Subsequently, the initiation complex, composed of the six viral factors, BMRF1, BSLF1, BBLF4, BBLF2/3, BALF5, and BALF2, is formed [61,62]. For our patients, apart from AIL26, *BZLF1* was highly expressed while *BRLF1* was little or not expressed. More, among the six mentioned genes, only *BMRF1* was expressed. Therefore, it seems to us that the virus is certainly in a latent state. This is why it is surprising to find such high levels of BNLF2a expression. BNLF2a expression has already been reported during latency, but mainly in carcinomas, especially GC [55] and non-small-cell lung carcinoma (NSCLC) [63], but not in lymphoma to our knowledge. It has even been proposed to call this latency, which includes the expression of EBNA-1, LMP-2, and BNLF2a, latency IIc [64]. This type of latency was carried by 11/14 of the AITLs we tested.

BNLF2a has been described to inhibit binding of peptides and ATP to the transporter activated peptide (TAP), resulting in down-regulation of the HLA class I proteins, thus blocking antigen presentation to cytotoxic T lymphocytes [65,66]. BNLF2a is expressed early in the productive lytic cycle to prevent infected cells from the recognition by CD8+ T cells sensitized to IE or E viral antigens [67], or in the pre-latent phase in B cells immediately following infection. A very high level of BNLF2a expression certainly makes it possible to protect the infected (tumor) cell from immune defenses. In addition, most AITL patients co-express *BCRF1*, a late gene encoding for the vIL-10 protein that has 80% homology with hIL-10. vIL-10 protects infected B cells from NK cell-mediated elimination, can inhibit CD4+ cell responses and the production of inflammatory cytokines [68], and promotes subsequent B cell proliferation and differentiation [69]. Jochum et al. [65] demonstrated that coexpression of *BNLF2a* and *BCRF1* contributes to the immune evasion of EBV during the very early phase of lytic infection. It can be assumed that, likewise, the simultaneous expression of these two proteins promotes the survival of infected cells and tumors.

In addition to *BCRF1*, five other late genes were expressed in almost all AITL samples. This strongly expressed gene cluster is made up of *BSRF1*, *BVRF1*, *BNRF1*, *BFRF3*, and *BOLF1*. Apart from *BCRF1* and *BNRF1*, which would play a role in cell immortalization, the others, as far as is known today, encode integument or capsid proteins. Clusters of lytic genes activated during latency have been described by others in NPC, GC, or BL [70], but the genes differ from those found here.

To better understand the behavior of EBV in lymphoma tissues, we decided to compare the results we obtained for our patients with those obtained for cell lines. We, therefore, studied the EBV transcriptome in the following lines: B95-8, four BL lines, two NK/TL lines, and four LCLs. Surprisingly, we did not find viral behavior specific to a pathology, just as LCLs do not all behave in the same way. Latency gene expression was particularly interesting, and EBNA-LP was highly expressed in all lines except MEC04. BARTs were somewhat more expressed in LCLs than in other lines, although the expression was low. As expected, our LCLs were found to be in latency III although, depending on the lines, the latency genes were expressed to varying degrees. The BL lines, that are derived from tumors exhibiting latency I, were mostly in latency I, while expressing *EBNA-LP* (Jijoye, Namalwa, P3HR1), or latency III for Raji, with a weak EBNA-3 expression. This latency change for Raji has already been reported and is due to growing conditions [38,71].

Interestingly, our lines showed much lower BART expression than patient tumors apart from the MEC04 line which had a very unique behavior. The MECO4 cell line was initially established from a patient with a fatal nasal NK-cell lymphoma at a leukemic stage [47]. MEC04 is the only line, among those studied here, strongly expressing BARTs as well as LMP2, while EBNA-1, LMP1, and EBNA-LP were weakly expressed and the other latency transcripts absent. Interestingly, Coppo et al. [47] reported that the STAT3 transcription factor is constitutively activated in the MEC04 cells line and suggested that STAT3 plays a primary role in nasal-NK/TL physiopathology. Recently, it was demonstrated that, in B lymphocytes, LMP2A, constitutively associated with Src family protein tyrosine kinases (PTKs) such as Syk, activates the phosphoinositide 3-kinase (PI3K)/Akt pathway [72]. Then, Bruton’s tyrosine kinase (BTK), regulated by PI3K, phosphorylates STAT3 which in turn activates cellular IL-10 [73]. The constitutive activation of STAT3 described by Coppo, therefore, appears to reflect the constitutive production of LMP2A by these cells. Results are very different from the other NK/TL line we studied, SNK6.

Among the most expressed genes in all lines was *BHRF1* which otherwise was very weakly expressed in patient samples. It encodes a protein whose role is poorly defined but which is a bcl-2 homolog and therefore exhibits anti-apoptotic properties essential for cell transformation [74,75]. EBV also encodes BHRF1 miRNAs which are consistently present in the early stages of infection. They restrict BHRF1 protein production and are also detectable in LCLs [76]. They contribute to B-cell transformation and proliferation [77]. Interestingly, EBV-miR-BHRF1-2 has been shown to downregulate *LMP2A*. In this study, the MEC04 line is the only one that expressed, albeit very weakly, *BHRF1* and *LMP2A* were, in contrast, highly expressed.

## 4. Materials and Methods

### 4.1. Production of Spontaneous LCLs

Spontaneously growing EBV-positive B-cell lines (LCLs), CoAN, DPL, KREB2, and MLEB2 cell lines, were established from the peripheral blood of four subjects whose characteristics are reported in Table 3. Lines were spontaneously established through the use of cyclosporin A [78] according to the protocol described by Sculley et al. [79].

### 4.2. Cell Lines and Culture

In addition to the LCLs established in our laboratory, seven other lines were used, the characteristics of which are mentioned in Table 3. B95-8 and the four BL lines (Jijoye, Namalwa, P3HR1, and Raji) were purchased from the ATCC (Catalog numbers CRL 1612—ECACC 85011419, CCL-87, CRL-1432, HTB-62, and CCL-86 respectively, Manassas, VA, USA). The extranodal NK/T cell lymphoma lines (MEC04 and SNK6) were kindly provided by Marion Travert (Inserm U955, Hôpital Henri Mondor, Créteil, France). All lines were grown in RPMI1640 medium with glutaMAX (ThermoFisher Scientific, Illkirch-Graffenstaden, France; catalog number 61870-010) supplemented with 10% fetal bovine serum (FBS; Eurobio Scientific, Les Ulis, France; catalog number CVFSVF00-0U) and 1% penicillin-gentamicin at 37 °C in a humidified 5% CO_2_ atmosphere. MEC04 and SNK6 cell lines were cultured under the same conditions and supplemented with 100 U/mL of human IL-2 (Sigma-Aldrich, Saint-Quentin Fallavier, France; catalog number I7908).

### 4.3. Patients

The population included in this study was composed of 14 patients with AITL and 21 other patients suffering from HL or B or T non-HL lymphoma (Table 4). All patients were initially diagnosed at Limoges University Hospital (France) after independent examination by two pathologists using WHO criteria [80]. Patients gave informed consent for the subsequent use of the samples taken and the study was retrospectively carried out on the lymphadenopathy used for the initial diagnosis. The study was approved by the Ethics Committee of the Institutional Review Board as part of ongoing studies, some of which being published soon. The patients were selected based on sample positivity for EBER, providing evidence of EBV infection.

### 4.4. EBER In Situ Hybridization

Detection of EBER1 by ISH was used to determine the presence of EBV allowing sample selection. Briefly, the formalin-fixed paraffin-embedded (FFPE) tissue sections were deparaffinized, rehydrated in a graded solution of xylene and alcohol, then deproteinized with proteinase K before incubation with the Ventana EBER 1 DNP Probe^®^ (Roche Diagnostics, Meylan, France; catalog number 760-1209) on the Benchmark XT automaton^TM^ (Roche Diagnostics). This was followed by staining with Ventana ISH iVIEW blue plus detection kit^®^ (Roche Diagnostics; catalog number 760-097) on the same apparatus.

For EBV positive samples, further work was performed on frozen material collected at the same time as the FFPE tissue.

### 4.5. EBV Typing

To determine the type of EBV present in each patient sample, we aligned sequences obtained against the unique EBNA-2/EBNA-3 regions of the prototype genomes of EBV type 1 (NC_007605) and type 2 (NC_009334) EBV. The prototype strain for which a greater number of reads matched for a given sample corresponded to the type of that sample.

### 4.6. RNA Extraction

Total RNA was extracted from cell lines and frozen biopsies by using the RNeasy mini kit^®^ (Qiagen, Les Ulis, France; catalog no. 74104) according to the manufacturer’s instructions and treated with DNase I by using RNase-free DNase set^®^ (Qiagen, catalog no. 79254). The frozen biopsies were previously disrupted and homogenized by adding lysis buffer and using the Precellys 24 tissue homogenizer^TM^ (Bertin Instruments, Montigny Le Bretonneux, France) while the lines were directly lysed by lysis buffer. Extracted RNA was re-suspended in RNAse-free water. RNA was then quantified and quality was determined using the Agilent RNA 6000 Nano Kit^®^ (Agilent Technologies, Les Ulis, France; catalog no. 5067-1511) on the Agilent 2100 Bioanalyzer^TM^ to obtain the RNA integrity number (RIN).

### 4.7. mRNA Enrichment

In brief, 700 ng high-quality total RNA, with a RIN ≥ 9, was used as input for each sample. Poly(A) mRNAs were selected by using oligo(dT) beads in a KAPA mRNA capture kit^®^ (Roche Diagnostics; catalog number 07962231001) according to the manufacturer’s protocol.

### 4.8. Probe Design for EBV Sequence Capture

The prototype genomes of EBV type 1 (NC_007605) and type 2 (NC_009334) EBV were used as references for designing EBV probes (Roche NimbleGen, Madison, WI, USA). Probes (100 to 120 bp) were designated to be overlapping and cover the entire viral genomes a minimum of 5 times, without matching the human hg19 genome (GRch38.p13), as determined by the SSAHA algorithm. Coverage for EBV-1 and EBV-2 genomes was estimated at 99.7% and 99.9%, respectively (a probe was considered to match the genome if there were fewer than five insertions, deletions, or substitutions of a single base between it and the genome). The vast majority of designated probes were unique, although some probes had a higher degree of multi-locus homology to increase coverage of all regions.

### 4.9. Sequencing by NGS

We used the high-throughput Illumina MiSeq^TM^ system (Illumina, Evry-Courcouronnes, France) to analyze the EBV transcriptome in the cell lines and selected patient samples (Bronner). The technique consists of an EBV mRNA capture from the poly(A) mRNAs by using NimbleGen SeqCap RNA Enrichment System^TM^ (Roche Diagnostics) according to the manufacturer’s protocol. Briefly, sample libraries were prepared using the KAPA Stranded RNA Library Preparation kit^®^ (Roche; catalog number 07962142001). Selected poly(A) mRNAs were first fragmented by enzyme digestion. Then first and second cDNA strands were synthetized and the obtained fragments were blunt-ended. A tailed and ligated to specific adapters from SeqCap Adapter kits^®^ A and B (Roche; catalog number 07141530001 and 07141548001, respectively). Subsequently, sample libraries were amplified for 11 cycles by a ligated mediation PCR (LM-PCR). At this step, a qualitative control using the High Sensitivity DNA kit^®^ (Agilent Technologies, catalog number 5067-4626) on the Agilent 2100 Bioanalyzer and a quantitative one using Qubit dsDNA HS Assay Kit^®^ (Invitrogen, catalog number Q32854) were performed. The amplified sample libraries were then pooled in equal molar quantities and hybridized to EBV biotinylated probes at 47 °C for 3 days consecutively using SeqCap Hybridization and Wash Kit^®^ (Roche; catalog number 05634261001). At that time, the hybridized fragments were recovered by magnetic streptavidin-beads (SeqCap Pure Capture Bead kit^®^, Roche; catalog number 06977952001) and enriched by 14 cycles of LM-PCR using SeqCap EZ Accessory kit v2^®^ (Roche; catalog number 07145594001). Size selection was performed during library preparation using a single Agencourt AMPure XP treatment from SeqCap Pure Capture Bead kit^®^, to remove DNA fragments below 200 bp. The final concentration was measured by the Agilent High Sensitivity DNA kit and libraries were finally 2 × 300-base paired-end sequenced on an Illumina MiSeq instrument^TM^ (ICM, Paris, France).

The sequencing of all cell lines was carried out in its entirety in triplicate.

### 4.10. Data Analysis

Data obtained were checked for quality using FastQC v 0.11.5 (Babraham bioinformatics, Cambridge, UK) and no trimming was done. Raw sequencing reads were aligned against hg19 (GRch38.p13), EBV1 (NC_007605.1), and EBV2 (NC_009334.1) reference genomes using bwa mem 0.7.17-r1188. Data were then quantified using featureCounts 1.6.0 (Walter and Eliza Hall bioinformatics, Melbourne, Australia. Data normalization and differential expression analysis were performed using a custom script to obtain transcripts per million (TPM). Samples were then averaged and put in log. Statistically, differentially-expressed genes were visualized with heatmaps (false discovery rate less than 0.05) using pheatmap (Raivo Kolde, University of Tartu, Tartu, Estonia), tidyverse (Hadley Wickham, University of Auckland, Auckland, New Zealand), hrbrthemes (Bob Rudis, Rapid7, Cambridge, USA), and viridis (Simon Garnier, New Jersey Institute of Technology, Newark, NJ, USA) R packages (built under R version 4.0.2).

## 5. Conclusions

In summary, our EBV transcriptome study on 14 AITL biopsies was compared to the results obtained for the other lymphomas tested and showed that BARTs were much more strongly and more frequently expressed for AITLs suggesting that they might play a role in this lymphoma. We have shown that AITLs exhibited a latency corresponding to the latency IIc described by Strong et al. [64] and that the simultaneous expression of BNLF2a and BCRF1 may allow infected cells to survive. Taken together, these results suggest the involvement of EBV in this pathology.

## Figures and Tables

**Table 1:** Results obtained for Epstein–Barr virus (EBV) gene expression by new generation sequencing (NGS) for the 11 cell lines and 35 patient samples studied.

Sample Name	Read Number	Mean Depth	EBV Type
B95-8	10,053,705	2010	EBV-1
CoAN	1,942,331	1251	EBV-1
DPL	3,490,546	1326	EBV-1
Jijoye	5,538,086	1586	EBV-1
KREB2	2,194,143	117	EBV-1
MECO4	894,293	61	EBV-2
MLEB2	2,351,251	537	EBV-1
Namalwa	2,697,676	778	EBV-2
P3HR1	3,622,454	698	EBV-1
Raji	1,993,483	65	EBV-1
SNK6	3,175,449	971	EBV-2
PTBL5	1,033,508	63	EBV-1
DLBCL3	1,071,114	42	EBV-1
AIL7	1,248,228	71	EBV-1
ARL2	3,254,648	873	EBV-1
AIL15	2,356,168	626	EBV-1
ALCL1	1,782,932	78	EBV-1
AIL25	3,143,910	751	EBV-1
AIL24	1,204,758	56	EBV-1
AIL27	889,930	45	EBV-1
AIL2	2,024,732	92	EBV-1
AIL11	910,244	47	EBV-1
DLBCL4	1,764,060	301	EBV-1
PTBL4	1,494,164	385	EBV-1
AIL3	1,066,140	46	EBV-1
CTCL1	4,930,412	792	EBV-1
PTBL6	1,009,426	180	EBV-1
AIL14	1,948,266	553	EBV-1
AIL16	1,214,934	63	EBV-1
NLPHL2	2,009,568	45	EBV-1
NS.CHL1	566,500	51	EBV-1
NS.CHL3	3,573,262	861	EBV-1
MC.CHL1	1,907,892	133	EBV-1
AIL22	1,168,146	46	EBV-1
NS.CHL2	3,669,640	48	EBV-1
AIL23	2,096,084	47	EBV-1
AIL26	1,360,172	42	EBV-1
PTCL.NOS4	1,752,840	45	EBV-1
AIL21	2,526,360	102	EBV-1
ALCL2	4,104,334	43	EBV-1
CTCL2	1,376,010	159	EBV-1
CTCL3	1,495,068	124	EBV-1
PTCL.NOS5	2,894,860	47	EBV-1
PTCL.NOS3	1,361,598	42	EBV-1
NLPHL1	2,610,342	74	EBV-1
DLBCL5	8,303,756	1027	EBV-1

**Table 2:** Characteristics of the cell lines used in this work.

Cell Line	Origin	Latency Type	Particularity
B95.8	Primary infection [31]	Lat III [32]	Deletion 139,724 bp to 151,554 bp: *OriLyt*—large part of miRNA *BART—LF-1*, *-2*, *-3* [31,33,34]
Jijoye	Endemic Burkitt’s lymphoma [35]	Lat III [36]	No deletion
Namalwa	Endemic Burkitt’s lymphoma [37]	Lat I [38] Lat III [36]	2 copies of EBV genome integrated into the human chromosome [39]
P3HR1	Burkitt’s lymphoma [40]	Lat I [36] Lat II [41] Atypical latency [42]	Derived from Jijoye—Deletion (33,355 bp to 40,163 bp): *EBNA-2*, part of *EBNA-LP*, part of *BHLF-1* [34,38,43]
Raji	Burkitt’s lymphoma [44]	Lat III [36] Lat I/Lat III (in vitro) [38]	Two deletions (99,126 bp to 102,118 bp and 163,978 bp to 166,635 bp): *EBNA-3C*, *BZLF2*, *BARF1*, *BALF1*, *BALF2* [45,46]
MEC04	NK/T lymphoma [47]	Lat II [47]	
SNK6	NK/T lymphoma [48]	Lat II [48]	EBNA-2 not expressed [49,50]

**Table 3:** Characteristics of lymphoblastoid cell line (LCL) origin patients.

Cell Line	Patient Age	Patient Sex	Patient Pathology
CoAN	61	F	Renal cell carcinoma and hepatocellular carcinoma
DPL	46	M	Cardiac AL λ amyloidosis
KREB2	64	M	Parsonage-Turner syndrome
MLEB2	66	M	Healthy subject

**Table 4:** Characteristics of the 14 AITLs and 21 other EBV-associated lymphomas patients included in this study.

Patient	Sex	Age at Diagnosis	Pathology According to WHO Criteria (2016)
AIL2	M	62	Angioimmunoblastic T-cell lymphoma
AIL3	M	80	Angioimmunoblastic T-cell lymphoma
AIL7	F	59	Angioimmunoblastic T-cell lymphoma
AIL11	M	59	Angioimmunoblastic T-cell lymphoma
AIL14	M	62	Angioimmunoblastic T-cell lymphoma
AIL15	M	67	Angioimmunoblastic T-cell lymphoma
AIL16	M	50	Angioimmunoblastic T-cell lymphoma
AIL21	M	79	Angioimmunoblastic T-cell lymphoma
AIL22	M	70	Angioimmunoblastic T-cell lymphoma
AIL23	M	67	Angioimmunoblastic T-cell lymphoma
AIL24	F	78	Angioimmunoblastic T-cell lymphoma
AIL25	M	59	Angioimmunoblastic T-cell lymphoma
AIL26	M	69	Angioimmunoblastic T-cell lymphoma
AIL27	F	69	Angioimmunoblastic T-cell lymphoma
PTCL-NOS3	M	81	Peripheral T-cell lymphoma, not otherwise specified
PTCL-NOS4	M	69	Peripheral T-cell lymphoma, not otherwise specified
PTCL-NOS5	F	80	Peripheral T-cell lymphoma, not otherwise specified
ALCL1	F	73	Anaplastic large T cell lymphoma
ALCL2	M	20	Anaplastic large T cell lymphoma
CTCL1	F	73	Cutaneous T cell lymphoma
CTCL2	M	63	Cutaneous T cell lymphoma
CTCL3	M	83	Cutaneous T cell lymphoma
NLPHL1	M	68	Nodular lymphocyte-predominant type Hodgkin’s lymphoma
NLPHL2	M	33	Nodular lymphocyte-predominant type Hodgkin’s lymphoma
NS-CHL1	M	20	Nodular sclerosis classical Hodgkin’s lymphoma
NS-CHL2	M	73	Nodular sclerosis classical Hodgkin’s lymphoma
NS-CHL3	F	67	Nodular sclerosis classical Hodgkin’s lymphoma
MC-CHL1	M	77	Mixed cellularity classical Hodgkin’s lymphoma
DLBCL3	F	59	Diffuse large B-cell lymphoma
DLBCL4	F	31	Diffuse large B-cell lymphoma
DLBCL5	F	59	Diffuse large B-cell lymphoma
PTBL4	F	52	Post-transplant B lymphoma
PTBL5	F	68	Post-transplant B lymphoma
PTBL6	M	57	Post-transplant B lymphoma
ARL2	F	76	Age-related lymphoma

## Data Availability

The data presented in this study are available in the article. EBV gene expression data generated in this study have been deposited as stated above (Section 2.1).
